# Use of a food frequency questionnaire in American Indian and Caucasian pregnant women: a validation study

**DOI:** 10.1186/1471-2458-5-135

**Published:** 2005-12-15

**Authors:** Heather J Baer, Robin E Blum, Helaine RH Rockett, Jill Leppert, Jane D Gardner, Carol W Suitor, Graham A Colditz

**Affiliations:** 1 Channing Laboratory, Department of Medicine, Brigham and Women's Hospital and Harvard Medical School, Boston, MA, USA; 2 Department of Epidemiology, Harvard School of Public Health, Boston, MA, USA; 3 North Dakota WIC Program, Department of Health, Bismarck, ND, USA; 4 Maternal and Child Health Consultant, Bedford, MA, USA; 5 Nutrition and Maternal and Child Health Consultant, Northfield, VT, USA; 6 Harvard Center for Cancer Prevention, Boston, MA, USA; 7 This author is deceased

## Abstract

**Background:**

Food frequency questionnaires (FFQs) have been validated in pregnant women, but few studies have focused specifically on low-income women and minorities. The purpose of this study was to examine the validity of the Harvard Service FFQ (HSFFQ) among low-income American Indian and Caucasian pregnant women.

**Methods:**

The 100-item HSFFQ was administered three times to a sample of pregnant women, and two sets of 24-hour recalls (six total) were collected at approximately 12 and 28 weeks of gestation. The sample included a total of 283 pregnant women who completed Phase 1 of the study and 246 women who completed Phase 2 of the study. Deattenuated Pearson correlation coefficients were used to compare intakes of 24 nutrients estimated from the second and third FFQ to average intakes estimated from the week-12 and week-28 sets of diet recalls.

**Results:**

Deattenuated correlations ranged from 0.09 (polyunsaturated fat) to 0.67 (calcium) for Phase 1 and from 0.27 (sucrose) to 0.63 (total fat) for Phase 2. Average deattenuated correlations for the two phases were 0.48 and 0.47, similar to those reported among other groups of pregnant women.

**Conclusion:**

The HSFFQ is a simple self-administered questionnaire that is useful in classifying low-income American Indian and Caucasian women according to relative dietary intake during pregnancy. Its use as a research tool in this population may provide important information about associations of nutrient intakes with pregnancy outcomes and may help to identify groups of women who would benefit most from nutritional interventions.

## Background

The importance of nutrition during pregnancy has long been appreciated. Overall nutritional adequacy, i.e., a sufficient supply of calories and protein, is a major determinant of weight gain during pregnancy [1,2]. Maternal intake of carbohydrates and protein [3], fatty acids [4], and micronutrients such as zinc [5-7], iron [2], magnesium [8-10], calcium [11], riboflavin [4], and vitamin C [12]may also have important effects on fetal growth and perinatal outcomes.

Food frequency questionnaires (FFQs) have been shown to be valuable tools for evaluating long-term dietary intake in the context of epidemiologic research [13], and some investigators have examined their reliability and validity among pregnant women [14-22]. While early studies focused on the validity of FFQs for assessing intakes of calories, protein, and a small number of micronutrients during pregnancy [21,22], several recent studies have examined a larger group of nutrients [15-17]. The results generally indicate that FFQs can be used to classify pregnant women according to their nutritional intake with a reasonable degree of accuracy, although this may vary according to the population and the number of food items on the instrument.

Low-income pregnant women may have poor nutritional status [23], and those with fewer years of formal education may have more difficulty completing a FFQ [24]. Food frequency questionnaires should be developed and tested among low-income pregnant women, as they could be useful for examining associations of nutrient intakes with pregnancy outcomes and for identifying groups of women who would benefit most from nutritional interventions. Block reported on the validity of two different FFQs (Block and Harvard) for assessing the diets of low-income pregnant, lactating, and non-pregnant, non-lactating women enrolled in the Women, Infants, and Children (WIC) Program in New York, California, Texas, and Ohio [19]. The Harvard Service FFQ (HSFFQ) has also been validated among low-income pregnant women attending prenatal health clinics in Massachusetts [16,22]. These studies, however, only included white, African American, and Hispanic women. The validity of FFQs for assessing dietary intake among pregnant women of other racial/ethnic backgrounds has yet to be determined.

The purpose of this study was to further expand the validity of the HSFFQ for use in the assessment of relative dietary intake among low-income American Indian and Caucasian pregnant women.

## Methods

### Subjects

**Figure 1:**
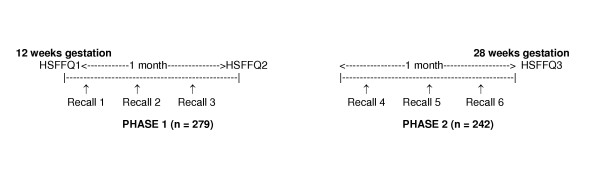
Time sequence of diet validation study conducted among pregnant women participating in North Dakota WIC.

With the review, approval and oversight of an internal review board, we sequentially recruited a sample of Caucasian and American Indian (Spirit Lake Sioux and Turtle Mountain Chippewa) pregnant women appearing in North Dakota WIC clinics in one of five towns for their initial prenatal nutrition counseling visit; several sites were selected based on having a high proportion of American Indians. Of the 317 women who were approached, 309 (97%) agreed to participate. Each woman signed a consent form at the routine WIC visit, confirming her willingness to participate. At the end of data collection, our sample included a total of 283 pregnant women (89% of those recruited) who completed Phase 1 of the study (set of three diet recalls and HSFFQ at 12 weeks of gestation) and 246 women (78% of those recruited) who completed Phase 2 of the study (set of three diet recalls and HSFFQ at 28 weeks of gestation) (Figure 1). Women who had implausibly high values for caloric intake on the FFQ (greater than 4,500 kilocalories per day) or were younger than age 16 were excluded, leaving a total of 279 and 242 participants for the Phase 1 and Phase 2 analyses, respectively.

### Harvard Service FFQ for pregnant women

**Figure 2:**
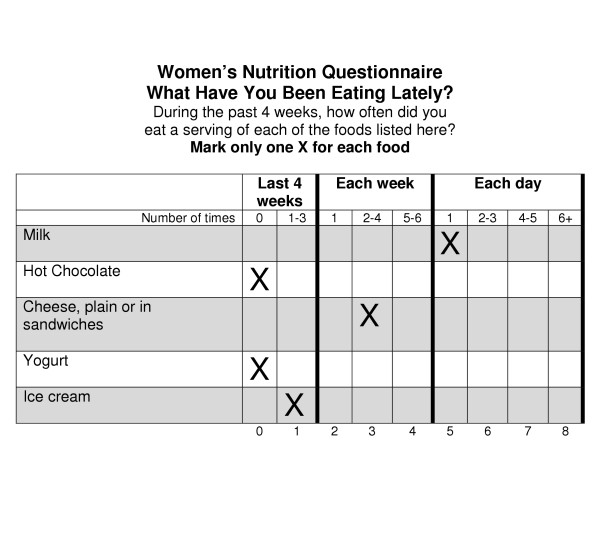
Sample of questions and layout of Harvard Service Food Frequency Questionnaire.

The Harvard Service FFQ was originally the Prenatal FFQ [22]. It consists of 100 items, including 84 foods and 16 questions about food habits and supplements, other services utilized (e.g., TANF, food stamps), and prenatal health (e.g., number of previous pregnancies, pregnancy side effects, medical problems or conditions) (sample food questions and layout shown in Figure 2). It was designed as a self-administered tool adapted from the FFQ developed and evaluated by Willett *et al*. [25,26]. Unlike the original Willett FFQ, however, which focuses on intake during the last year, the HSFFQ is designed to categorize pregnant women by their intake over the past four weeks. It is completed on a paper form or entered directly into a computer program; this study used the paper form exclusively. The HSFFQ used in North Dakota was modified to include deer and fry bread, which are foods more commonly consumed by the local populations. Portion sizes used with the HSFFQ are derived from national data [27], and the database used for the nutrient analysis was a specifically designed program harvardsffq.022101 (February 21, 2001). The foundation of this database is the U.S. Department of Agriculture Nutrient Database for Standard Reference [27-29], with additional information from McCance and Widdowson's *The Composition of Foods *[30,31], journals, and manufacturers.

### Collection of FFQ and 24-hour diet recalls

The FFQ was completed by each woman for the first time at a routine 12-week prenatal WIC visit (HSFFQ1). Following the first visit, three 24-hour recalls were completed over the subsequent month, approximately every seven to ten days (week-12 set of diet recalls). In general, two recalls were taken on weekdays and one recall on the weekend. The FFQ was completed a second time (HSFFQ2) after the week-12 set of diet recalls and then a third time (HSFFQ3) at a subsequent routine 28-week prenatal WIC visit, following the collection of a second set of three 24-hour recalls over the preceding month (week-28 set of diet recalls).

The 24-hour diet recalls were administered by telephone or in person. Each woman's intake was entered directly into the computer by a registered dietitian working for North Dakota WIC and familiar with this population. All seven dietitians attended a five-hour training session during which they were tested on the reliability of their recalls. Standard models were used to establish the sizes of small, medium, and large portions if measurements could not be given, and any unusual intake was noted on the recall. Data were checked by a local study coordinator, also a registered dietitian, in North Dakota and then sent to our offices in Boston where they were again checked for plausible intake by the supervising research nutritionist; the protocol and editing guidelines were established prior to the beginning of the study. The nutrient calculations for the 24-hour recalls were performed with the Minnesota Nutrient Data System software, developed by the Nutrition Coordinating Center, University of Minnesota (Minneapolis, MN), Food Database Version 6A, Nutrient Database Version S21.

### Statistical analysis

All nutrient intakes were log-transformed prior to analysis to improve their normality. Energy-adjusted intakes were computed using the residual method, where each nutrient is regressed on total calories, and the population mean was then added back to the calculated residuals [32]. Mean nutrient intakes for the diet recalls were calculated by averaging the energy-adjusted intakes from the three recalls for each time period. Pearson correlation coefficients were used to compare energy-adjusted intakes estimated from the FFQ and the diet recalls for each phase of the study (Phase 1: HSFFQ2 vs. week-12 set of diet recalls, Phase 2: HSFFQ3 vs. week-28 set of diet recalls). Deattenuated correlation coefficients were also calculated in order to correct for random within-person variation in the 24-hour recalls [33, 34]. These correlations were first computed for all participants and then separately by ethnicity (American Indian and Caucasian), percentage of poverty level (≤ 100% and 101–185%), and number of previous livebirths (0, 1, and ≥ 2). In addition, energy-adjusted intakes from the HSFFQ and the diet recalls were divided into quartiles and jointly classified to assess the percent agreement between categorizations based on the two methods (i.e., the percent of women ranked in the same category according to both measures). Pearson correlation coefficients were used to compare energy-adjusted intakes from the week-12 and the week-28 sets of recalls for participants who completed the entire study. Intakes from the recalls for these two time periods were also jointly classified according to quartiles. All statistical analyses were performed using SAS (Release 6.12; SAS Institute, Cary, NC).

## Results

Study participants ranged in age from 16 to 40 years, with a mean age of 24. Of the 279 women who completed Phase 1, 104 (37%) were American Indian and 175 (63%) were Caucasian. Sixty-three percent of women who completed Phase 1 were at 100% or less of the poverty level, and 43% had no previous livebirths. Population characteristics were similar for women who completed Phase 1, those who completed Phase 2, and those who completed the entire study (Table 1).

**Table 1:** Characteristics of low-income pregnant women in a validation study of the Harvard Service FFQ (HSFFQ)

**Characteristic**	**Phase 1: Three week-12 recalls + HSFFQ2 (n = 279) ^a^**	**Phase 2: Three week-28 recalls + HSFFQ3 (n = 242) ^b^**	**Complete study: Three week-12 recalls + three week-28 recalls + HSFFQ2 + HSFFQ3 (n = 237) ^c^**
Mean age (range)	24 (16–40)	24 (16–39)	24 (16–39)
	n (%)	n (%)	n (%)
Ethnicity			
American Indian	104 (37)	88 (36)	82 (35)
Caucasian	175 (63)	154 (64)	154 (65) ^d^
Percent poverty level			
≤ 100%	176 (63)	151 (62)	147 (62)
101 to 133%	44 (16)	41 (17)	41 (17)
134 to 185%	56 (20) ^e^	50 (21)	49 (21)
Number of previous livebirths			
0	120 (43)	100 (41)	98 (41)
1	78 (28)	69 (29)	67 (28)
2	46 (16)	43 (18)	43 (18)
3	23 (8)	22 (9)	22 (9)
≥ 4	11 (4) ^f^	8 (3)	7 (3)

Some percentages do not sum to exactly 100% due to rounding

^a ^Excluded if calories > 4500 (n = 3) or age < 16 (n = 1) on the HSFFQ2

^b ^Excluded if calories > 4500 (n = 2) or age < 16 (n = 2) on the HSFFQ3

^c ^Excluded if calories > 4500 (n = 5) or age < 16 (n = 3) on the HSFFQ2 or HSFFQ3

^d ^Excluding 1 participant with discordant ethnicity reported on the HSFFQ2 and HSFFQ3

^e ^Percent poverty level missing for 3 participants

^f ^Number previous livebirths missing for 1 participant

Pearson correlation coefficients for energy-adjusted nutrient intakes estimated from the FFQ and the 24-hour diet recalls for all participants ranged from 0.03 to 0.52 at 12 weeks and from 0.21 to 0.48 at 28 weeks; the average correlations for both Phase 1 and Phase 2 were 0.35 (Tables 2 and 3). After correction for random within-person variation, the deattenuated correlations for all participants ranged from 0.09 for polyunsaturated fat (95% CI: -0.13, 0.31) to 0.67 for calcium (95% CI: 0.53, 0.77) for Phase 1 and from 0.27 for sucrose (95% CI: 0.09, 0.43) to 0.63 for total fat (95% CI: 0.47, 0.75) for Phase 2. Average deattenuated correlations for the two phases were 0.48 and 0.47, respectively. Sixteen nutrients had deattenuated correlations of 0.50 or greater at 12 weeks and 11 nutrients reached correlations of 0.50 or greater at 28 weeks (Tables 2 and 3). The greatest percentage agreement for being ranked in the lowest quartile by both methods was 59% at 12 weeks (vitamin B2) and 57% at 28 weeks (zinc). The greatest percentage agreement for being ranked in the highest quartile by both methods was 49% at 12 weeks (calcium and phosphorus) and 50% at 28 weeks (vitamin B6).

**Table 2:** Descriptive statistics and Pearson correlation coefficients for three week-12 diet recalls and the HSFFQ2 ^a^

	**Overall**				**By ethnicity**		**By poverty level**	
	**All Phase 1 participants**				**American Indian**	**Caucasian**	**≤ 100%**	**101–185%**
**Nutrient**	**Mean of week-12 recalls (SD)**	**Mean of HSFFQ2 (SD)**	**Adjusted r ^b^**	**Deattenuated r ^c ^(95%CI)**	**Deattenuated r ^c ^(95%CI)**	**Deattenuated r ^c ^(95% CI)**	**Deattenuated r ^c ^(95% CI)**	**Deattenuated r ^c ^(95% CI)**
Calories (kcal)	2139 (629)	1927 (716)	NA	NA	NA	NA	NA	NA
Carbohydrates (g)	280 (83)	240 (92)	0.15	0.22 (0.02, 0.41)	0.15 (-0.13, 0.41)	0.30 (-0.03, 0.57)	0.26 (0.03, 0.46)	0.23 (-0.37, 0.69)
Protein (g)	82 (27)	77 (32)	0.31	0.51 (0.31, 0.67)	0.44 (0.05, 0.72)	0.54 (0.29, 0.73)	0.46 (0.21, 0.66)	0.64 (0.27, 0.85)
Dietary fiber (g)	15 (6)	13 (7)	0.19	0.27 (0.10, 0.42)	0.26 (-0.02, 0.50)	0.36 (0.14, 0.54)	0.30 (0.10, 0.48)	0.32 (0.02, 0.57)
Total fat (g)	79 (31)	77 (33)	0.28	0.41 (0.24, 0.56)	0.42 (0.10, 0.67)	0.39 (0.18, 0.57)	0.47 (0.24, 0.65)	0.32 (0.03, 0.55)
Saturated fat (g)	29 (12)	30 (13)	0.36	0.50 (0.33, 0.64)	0.62 (0.28, 0.82)	0.45 (0.25, 0.62)	0.62 (0.38, 0.78)	0.33 (0.06, 0.55)
Monounsaturated fat (g)	30 (11)	29 (13)	0.27	0.39 (0.21, 0.54)	0.37 (0.02, 0.65)	0.37 (0.15, 0.55)	0.42 (0.19, 0.60)	0.34 (0.04, 0.58)
Polyunsaturated fat (g)	15 (8)	12 (6)	0.03	0.09 (-0.13, 0.31)	0.08 (-0.28, 0.42)	0.10 (-0.18, 0.37)	0.17 (-0.12, 0.44)	-
Omega 3 fatty acids (g)	0.05 (0.10)	0.04 (0.05)	0.25	0.33 (0.13, 0.50)	0.22 (-0.05, 0.47)	0.42 (0.10, 0.66)	0.36 (0.11, 0.57)	0.27 (-0.07, 0.55)
Percent energy from fat	33 (6)	36 (6)	NA	NA	NA	NA	NA	NA
Cholesterol	268 (148)	247 (128)	0.37	0.57 (0.38, 0.72)	0.43 (0.10, 0.68)	0.63 (0.36, 0.80)	0.63 (0.37, 0.80)	0.47 (0.15, 0.71)
Sucrose	43 (23)	27 (15)	0.26	0.36 (0.19, 0.52)	0.16 (-0.14, 0.43)	0.46 (0.24, 0.63)	0.34 (0.12, 0.53)	0.41 (0.13, 0.64)
Calcium	1320 (560)	1387 (566)	0.52	0.67 (0.53, 0.77)	0.55 (0.29, 0.73)	0.60 (0.39, 0.76)	0.69 (0.47, 0.83)	0.58 (0.36, 0.74)
Iron (mg)	55 (27)	58 (23)	0.42	0.51 (0.38, 0.62)	0.40 (0.18, 0.59)	0.58 (0.41, 0.71)	0.50 (0.34, 0.64)	0.50 (0.25, 0.68)
Magnesium (mg)	290 (117)	257 (101)	0.35	0.45 (0.30, 0.58)	0.35 (0.07, 0.58)	0.47 (0.27, 0.64)	0.35 (0.16, 0.52)	0.70 (0.36, 0.87)
Phosphorus (mg)	1473 (521)	1485 (572)	0.45	0.63 (0.45, 0.75)	0.42 (0.12, 0.66)	0.65 (0.40, 0.81)	0.57 (0.33, 0.74)	0.68 (0.38, 0.85)
Zinc (mg)	28 (11)	30 (11)	0.43	0.53 (0.40, 0.64)	0.44 (0.20, 0.63)	0.59 (0.40, 0.72)	0.56 (0.40, 0.69)	0.46 (0.21, 0.65)
Vitamin A (IU)	9709 (7274)	10602 (5001)	0.33	0.56 (0.39, 0.69)	0.69 (0.33, 0.87)	0.43 (0.18, 0.62)	0.63 (0.40, 0.79)	0.30 (-0.01, 0.55)
Vitamin E (mg)	17 (9)	13 (5)	0.37	0.52 (0.38, 0.64)	0.43 (0.18, 0.64)	0.58 (0.37, 0.73)	0.51 (0.33, 0.65)	0.51 (0.21, 0.72)
Vitamin C (mg)	192 (94)	227 (109)	0.49	0.64 (0.49, 0.75)	0.73 (0.41, 0.89)	0.56 (0.38, 0.70)	0.63 (0.44, 0.76)	0.59 (0.33, 0.77)
Vitamin B1 (mg)	3 (2)	3 (1)	0.41	0.50 (0.37, 0.61)	0.55 (0.30, 0.73)	0.45 (0.28, 0.59)	0.51 (0.34, 0.65)	0.43 (0.21, 0.61)
Vitamin B2 (mg)	4 (2)	4 (1)	0.51	0.61 (0.49, 0.71)	0.62 (0.39, 0.77)	0.52 (0.35, 0.65)	0.63 (0.46, 0.75)	0.52 (0.31, 0.68)
Niacin (mg)	36 (11)	33 (12)	0.37	0.50 (0.35, 0.62)	0.48 (0.22, 0.67)	0.55 (0.34, 0.70)	0.45 (0.27, 0.60)	0.65 (0.31, 0.84)
Folate (mcg)	840 (358)	929 (350)	0.45	0.57 (0.44, 0.67)	0.52 (0.30, 0.69)	0.59 (0.41, 0.72)	0.58 (0.42, 0.70)	0.51 (0.25, 0.70)
Vitamin B6 (mg)	4 (2)	4 (1)	0.43	0.53 (0.40, 0.64)	0.50 (0.26, 0.69)	0.54 (0.37, 0.68)	0.51 (0.34, 0.65)	0.53 (0.31, 0.70)
Vitamin B12 (mcg)	8 (4)	8 (4)	0.40	0.53 (0.37, 0.67)	0.55 (0.16, 0.79)	0.55 (0.33, 0.71)	0.56 (0.35, 0.72)	0.54 (0.21, 0.75)
	Average correlation:		0.35	0.48	0.46	0.51	0.51	0.48

SD, standard deviation; CI, confidence interval

^a ^Excluded if calories > 4500 (n = 3) or age < 16 (n = 1) on the HSFFQ2 (n = 279)

^b ^Adjusted for total energy intake

^c ^Adjusted for total energy intake and corrected for random within-person variation; omega 3 fatty acids and polyunsaturated fat not included in average deattenuated correlations stratified by ethnicity and poverty level

**Table 3:** Descriptive statistics and Pearson correlation coefficients for three week-28 diet recalls and the HSFFQ3 ^a^

	**Overall**				**By ethnicity**		**By poverty level**	
	**All Phase 2 participants**				**American Indian**	**Caucasian**	**≤ 100%**	**101–185%**
**Nutrient**	**Mean of week-28 recalls (SD)**	**Mean of HSFFQ3 (SD)**	**Adjusted r ^b^**	**Deattenuated r ^c ^(95%CI)**	**Deattenuated r ^c ^(95%CI)**	**Deattenuated r ^c ^(95% CI)**	**Deattenuated r ^c ^(95% CI)**	**Deattenuated r ^c ^(95% CI)**
Calories (kcal)	2162 (592)	2010 (735)	NA	NA	NA	NA	NA	NA
Carbohydrates (g)	286 (79)	246 (89)	0.26	0.35 (0.18, 0.50)	0.23 (-0.06, 0.49)	0.41 (0.21, 0.58)	0.43 (0.22, 0.60)	0.17 (-0.11, 0.42)
Protein (g)	81 (26)	82 (31)	0.29	0.40 (0.23, 0.54)	0.10 (-0.18, 0.37)	0.55 (0.34, 0.71)	0.45 (0.24, 0.63)	0.28 (-0.01, 0.53)
Dietary fiber (g)	15 (6)	13 (6)	0.31	0.43 (0.27, 0.57)	0.02 (-0.30, 0.33)	0.60 (0.41, 0.74)	0.51 (0.28, 0.69)	0.35 (0.10, 0.56)
Total fat (g)	80 (28)	81 (35)	0.48	0.63 (0.47, 0.75)	0.51 (0.18, 0.74)	0.66 (0.48, 0.79)	0.68 (0.50, 0.81)	0.45 (0.14, 0.68)
Saturated fat (g)	29 (11)	32 (13)	0.46	0.62 (0.46, 0.74)	0.42 (0.14, 0.64)	0.70 (0.49, 0.83)	0.65 (0.46, 0.78)	0.52 (0.23, 0.72)
Monounsaturated fat (g)	30 (12)	31 (13)	0.41	0.57 (0.38, 0.70)	0.53 (0.12, 0.79)	0.54 (0.33, 0.70)	0.64 (0.42, 0.80)	0.35 (0.04, 0.60)
Polyunsaturated fat (g)	14 (6)	13 (6)	0.21	0.33 (0.10, 0.52)	0.37 (-0.08, 0.69)	0.30 (0.03, 0.52)	0.41 (0.14, 0.63)	0.15 (-0.24, 0.50)
Omega 3 fatty acids (g)	0.04 (0.10)	0.04 (0.05)	0.26	0.52 (-0.12, 0.86)	0.33 (-0.14, 0.67)	-	0.67 (-0.43, 0.97)	0.35 (-0.70, 0.92)
Percent energy from fat	33 (5)	36 (5)	NA	NA	NA	NA	NA	NA
Cholesterol	267 (148)	265 (128)	0.29	0.47 (0.24, 0.64)	0.35 (0.03, 0.61)	0.45 (-0.02, 0.76)	0.47 (0.17, 0.69)	0.45 (0.07, 0.72)
Sucrose	47 (23)	29 (13)	0.23	0.27 (0.09, 0.43)	0.23 (-0.07, 0.50)	0.27 (0.06, 0.47)	0.21 (-0.01, 0.40)	0.50 (0.17, 0.72)
Calcium	1364 (639)	1464 (502)	0.39	0.48 (0.33, 0.60)	0.39 (0.07, 0.63)	0.39 (0.20, 0.55)	0.47 (0.27, 0.62)	0.52 (0.28, 0.70)
Iron (mg)	61 (30)	59 (21)	0.44	0.51 (0.38, 0.63)	0.51 (0.25, 0.70)	0.50 (0.33, 0.64)	0.68 (0.50, 0.80)	0.35 (0.11, 0.55)
Magnesium (mg)	291 (105)	271 (98)	0.39	0.50 (0.35, 0.62)	0.43 (0.08, 0.69)	0.55 (0.36, 0.69)	0.49 (0.29, 0.66)	0.52 (0.28, 0.70)
Phosphorus (mg)	1475 (517)	1565 (537)	0.36	0.46 (0.30, 0.59)	0.26 (-0.04, 0.52)	0.42 (0.22, 0.59)	0.41 (0.20, 0.59)	0.54 (0.29, 0.73)
Zinc (mg)	30 (13)	31 (10)	0.48	0.60 (0.46, 0.72)	0.55 (0.27, 0.75)	0.58 (0.41, 0.71)	0.79 (0.61, 0.89)	0.41 (0.15, 0.61)
Vitamin A (IU)	11926 (9178)	10943 (4962)	0.25	0.35 (0.19, 0.50)	0.32 (0.01, 0.57)	0.36 (0.15, 0.53)	0.42 (0.19, 0.60)	0.28 (0.03, 0.50)
Vitamin E (mg)	16 (7)	13 (4)	0.34	0.44 (0.28, 0.58)	0.38 (0.07, 0.63)	0.45 (0.27, 0.61)	0.62 (0.42, 0.76)	0.26 (-0.01, 0.49)
Vitamin C (mg)	189 (84)	220 (88)	0.31	0.40 (0.24, 0.54)	0.26 (-0.06, 0.52)	0.41 (0.21, 0.57)	0.42 (0.21, 0.59)	0.37 (0.10, 0.58)
Vitamin B1 (mg)	3 (1)	3 (1)	0.38	0.52 (0.37, 0.65)	0.51 (0.22, 0.72)	0.51 (0.32, 0.66)	0.60 (0.41, 0.74)	0.43 (0.16, 0.65)
Vitamin B2 (mg)	4 (1)	4 (1)	0.44	0.56 (0.42, 0.68)	0.58 (0.30, 0.77)	0.52 (0.34, 0.67)	0.66 (0.48, 0.79)	0.46 (0.22, 0.65)
Niacin (mg)	36 (11)	34 (10)	0.32	0.45 (0.29, 0.58)	0.38 (0.12, 0.60)	0.51 (0.30, 0.68)	0.54 (0.35, 0.69)	0.31 (0.03, 0.54)
Folate (mcg)	888 (372)	936 (302)	0.45	0.55 (0.40, 0.66)	0.53 (0.23, 0.74)	0.52 (0.34, 0.66)	0.66 (0.47, 0.79)	0.42 (0.18, 0.61)
Vitamin B6 (mg)	4 (1)	4 (1)	0.41	0.52 (0.37, 0.64)	0.40 (0.13, 0.62)	0.54 (0.36, 0.69)	0.60 (0.41, 0.74)	0.42 (0.17, 0.62)
Vitamin B12 (mcg)	9 (4)	9 (4)	0.31	0.46 (0.30, 0.60)	0.27 (-0.07, 0.55)	0.53 (0.33, 0.69)	0.48 (0.26, 0.66)	0.42 (0.15, 0.64)
	Average correlation:		0.35	0.47	0.37	0.50	0.54	0.40

SD, standard deviation; CI, confidence interval

^a ^Excluded if calories > 4500 (n = 2) or age < 16 (n = 2) on the HSFFQ3 (n = 242)

^b ^Adjusted for total energy intake

^c ^Adjusted for total energy intake and corrected for random within-person variation; omega 3 fatty acids and polyunsaturated fat not included in average deattenuated correlations stratified by ethnicity and poverty level

Deattenuated correlation coefficients for most nutrients were fairly similar across subgroups when computed separately by ethnicity and poverty level (Tables 2 and 3). However, average deattenuated correlations comparing nutrient intakes estimated from the FFQ and the diet recalls were slightly lower for American Indians than for Caucasians (0.46 vs. 0.51 for Phase 1, 0.37 vs. 0.50 for Phase 2) and slightly higher for women at 100% or less of the poverty level than for those at 101% or greater (0.51 vs. 0.48 for Phase 1, 0.54 vs. 0.40 for Phase 2). Deattenuated correlation coefficients were also higher for women with one previous livebirth than for those with none and those with two or more previous livebirths. The average deattenuated correlations comparing nutrient intakes estimated from the FFQ and the diet recalls for Phase 1 were 0.47 for those with no previous livebirths, 0.58 for those with one previous livebirth, and 0.41 for those with two or more previous livebirths, and these were similar for Phase 2 (data not shown). Deattenuated correlation coefficients for omega 3 fatty acids and polyunsaturated fat could not be computed in some subgroups due to small numbers of participants and high within-person variation; hence, these nutrients were not included in average deattenuated correlations for any of the subgroups.

When nutrient intakes estimated from the week-12 set of recalls and the week-28 set of recalls were compared to one another to examine the stability of diet over the course of pregnancy, correlations between energy-adjusted intakes at 12 weeks and 28 weeks ranged from -0.02 for omega 3 fatty acids to 0.51 for calcium, with an average correlation of 0.33 (Table 4). The correlations between intakes at 12 weeks and 28 weeks were also similar for most nutrients within subgroups defined by ethnicity and poverty level. Nutrients with the greatest percentage agreement for the extreme quartiles at 12 weeks and 28 weeks were vitamin B2 and folate, with 54% agreement for the lowest quartile, and dietary fiber, with 53% agreement for the highest quartile (Table 5).

**Table 4:** Medians and Pearson correlation coefficients for three week-12 diet recalls and three week-28 diet recalls ^a^

	**Overall**			**By ethnicity**		**By poverty level**	
	**All participants**			**American Indian**	**Caucasian**	**≤ 100%**	**101–185%**
**Nutrient**	**Median** **of week-12** **recalls**	**Median** **of week-28** **recalls**	**Adjusted r ^b^**	**Adjusted r ^b^**	**Adjusted r ^b^**	**Adjusted r ^b^**	**Adjusted r ^b^**
Calories (kcal)	2122	2084	NA	NA	NA	NA	NA
Carbohydrates (g)	282	281	0.28	0.30	0.26	0.30	0.22
Protein (g)	81	78	0.26	0.24	0.29	0.25	0.30
Dietary fiber (g)	14	14	0.42	0.21	0.49	0.39	0.44
Total fat (g)	78	76	0.33	0.20	0.38	0.32	0.38
Saturated fat (g)	28	28	0.46	0.36	0.49	0.46	0.48
Mono unsaturated fat (g)	30	28	0.31	0.21	0.34	0.31	0.31
Poly unsaturated fat (g)	14	13	0.05	0.02	0.09	0.06	0.05
Omega 3 fatty acids (g)	0.02	0.02	-0.02	-0.002	-0.02	-0.11	0.13
Percent energy from fat	33	33	NA	NA	NA	NA	NA
Cholesterol	241	234	0.37	0.42	0.24	0.38	0.36
Sucrose	39	44	0.42	0.54	0.36	0.39	0.47
Calcium	1302	1278	0.51	0.47	0.48	0.45	0.59
Iron (mg)	62	70	0.46	0.38	0.54	0.44	0.53
Magnesium (mg)	276	276	0.48	0.33	0.54	0.47	0.48
Phosphorus (mg)	1487	1422	0.40	0.34	0.40	0.35	0.45
Zinc (mg)	32	32	0.43	0.33	0.51	0.41	0.46
Vitamin A (IU)	8515	9275	0.20	0.37	0.09	0.17	0.26
Vitamin E (mg)	16	16	0.35	0.21	0.44	0.37	0.32
Vitamin C (mg)	179	184	0.37	0.34	0.41	0.43	0.25
Vitamin B1 (mg)	3	3	0.29	0.31	0.27	0.38	0.16
Vitamin B2 (mg)	4	4	0.27	0.35	0.18	0.34	0.16
Niacin (mg)	37	36	0.38	0.35	0.39	0.39	0.39
Folate (mcg)	968	950	0.38	0.32	0.45	0.38	0.42
Vitamin B6 (mg)	4	4	0.27	0.34	0.20	0.37	0.13
Vitamin B12 (mcg)	8	8	0.26	0.16	0.27	0.25	0.28
	Average correlation:		0.33	0.30	0.34	0.33	0.33

SD, standard deviation; CI, confidence interval

^a ^Excluded if calories > 4500 (n = 5) or age < 16 (n = 3) on the HSFFQ2 or HSFFQ3 (n = 237)

^b ^Adjusted for total energy intake

**Table 5:** Joint classification of nutrient intakes from three week-12 diet recalls and three week-28 diet recalls ^a^

	**Percentage in same quartile of intake for phase 1 and phase 2 recalls ^b^**			
	
**Nutrient**	**Quartile 1 (low)**	**Quartile 2**	**Quartile 3**	**Quartile 4 (high)**
Carbohydrates (g)	44	32	30	36
Protein (g)	31	29	22	34
Dietary fiber (g)	31	34	27	53
Total fat (g)	37	27	38	31
Saturated fat (g)	39	29	32	46
Mono unsaturated fat (g)	39	22	38	36
Poly unsaturated fat (g)	27	29	30	25
Omega 3 fatty acids (g)	27	29	32	27
Cholesterol	29	29	35	44
Sucrose	47	29	27	41
Calcium	49	24	33	44
Iron (mg)	51	34	40	41
Magnesium (mg)	42	32	38	51
Phosphorus (mg)	39	25	27	44
Zinc (mg)	51	31	43	42
Vitamin A (IU)	41	25	38	37
Vitamin E (mg)	42	25	33	37
Vitamin C (mg)	51	32	25	42
Vitamin B1 (mg)	53	29	38	39
Vitamin B2 (mg)	54	29	30	44
Niacin (mg)	46	32	37	36
Folate (mcg)	54	32	42	41
Vitamin B6 (mg)	51	32	37	32
Vitamin B12 (mcg)	41	20	23	31

^a ^Excluded if calories > 4500 (n = 5) or age < 16 (n = 3) on the HSFFQ2 or HSFFQ3 (n = 237)

^b ^Nutrient intakes adjusted for total energy intake before quartile classification

## Discussion

The results of this study indicate that the Harvard Service FFQ can provide a reasonable assessment of relative nutritional intake among low-income American Indian and Caucasian women during pregnancy. In validation studies conducted in other adult populations, average correlations between nutrient intakes from FFQs and diet recalls have typically been in the range of 0.6 to 0.7 [13], which are slightly higher than those observed in this study. However, it is not surprising that correlations would be somewhat lower among pregnant women, given that diet during pregnancy may be less stable than during other time periods. The correlations in this study are comparable with those reported among other groups of pregnant women and suggest that the HSFFQ may be a useful tool for classifying these women according to nutritional intake.

Food frequency questionnaires are generally considered to be the most appropriate method for assessing diet in the context of epidemiologic studies. Besides offering practical advantages over more expensive and time-consuming methods, such as 24-hour diet recalls and food records, FFQs can provide better assessments of usual intake over longer periods of time, such as weeks or months, rather than a single day [13]. Because pregnant women's diets may change within each trimester, the assessment of diet during the past month is likely to be more accurate than during the past year. Previous studies have examined the validity of FFQs among pregnant women. A community-based study of 569 women in the United Kingdom reported correlations ranging from 0.27 to 0.55 for 20 energy-adjusted nutrients assessed by a 100-item FFQ and four-day food diaries at 15 weeks of pregnancy [18]. Brown *et al*. reported an average deattenuated correlation of 0.45 for changes in energy-adjusted intakes of 15 nutrients during pregnancy among 56 well-educated white women, assessed by a FFQ and by four-day food records [17]. In a study of 113 Finnish women in their third trimester of pregnancy, Erkkola *et al*. obtained an average deattenuated correlation coefficient of 0.53 for 45 nutrients assessed by a 181-item FFQ and two five-day food records [15].

Low-income pregnant women may have poorer nutritional status than other pregnant women. A recent study reported that diet quality among 2063 pregnant women in North Carolina who were between 24 and 29 weeks of gestation was significantly lower among poorer women and those with less education [23]. In addition, Kristal *et al*. assessed the validity of a FFQ among 1015 postmenopausal women according to race/ethnicity and level of education, and their results indicated that the validity of the FFQ increased with years of education and was higher among whites compared to blacks [24].

Only a few studies have evaluated the validity of FFQs among low-income pregnant women. Suitor *et al*. examined the validity of the HSFFQ for the assessment of total calories and seven nutrients (protein, calcium, iron, zinc, and vitamins A, B6, and C) among 95 low-income pregnant women in Massachusetts, comparing intakes estimated from the FFQ to those estimated from three 24-hour diet recalls [22]. With the exception of vitamin A, all of the deattenuated correlation coefficients for nutrient intakes were greater than 0.50. Wei *et al*. extended these results in the same group of women by examining the validity of the HSFFQ for the assessment of 17 additional nutrients; they reported a mean deattenuated correlation of 0.47, with correlations ranging from 0.03 for vitamin B12 to 0.90 for zinc [16]. In a study among African American, Hispanic, and white pregnant women in the WIC Program in four states, deattenuated correlation coefficients between energy-adjusted intakes assessed by the HSFFQ or the Block FFQ compared to 24-hour diet recalls were reported for five nutrients [19]. The highest correlation for the HSFFQ was for calcium (0.47) and the lowest correlation was for vitamin C (0.08); the correlations for the Block FFQ were very similar.

These previous studies did not examine whether the performance of the HSFFQ varies according to stage of pregnancy. The results of the present study indicate that the HSFFQ has similar validity during the first and second trimesters, as shown by the average deattenuated correlation coefficients of 0.48 and 0.47 for the week-12 and week-28 FFQs compared to the 24-hour diet recalls. These correlations are comparable to those observed among other groups of pregnant women [15-17,22]. This study is also unique because it examined the validity of a FFQ among American Indian pregnant women. Although the observed correlations were slightly lower for American Indians than for Caucasians, the results still suggest that the HSFFQ can be used in a variety of populations to assess nutritional intake during pregnancy.

Sixteen of the nutrients examined had deattenuated correlations of 0.50 or greater between energy-adjusted intakes estimated from the FFQ and the diet recalls at 12 weeks, and 11 nutrients had correlations of this magnitude at 28 weeks. In contrast, the correlation for polyunsaturated fat was very low in Phase 1 (deattenuated r = 0.09), and the 95% confidence intervals for polyunsaturated fat and omega 3 fatty acids included negative values either in Phase 1 or Phase 2. This was a particular problem in the stratified analyses and may have been a consequence of small sample size, high day-to-day variation in intake of some nutrients among individuals in this population, and reduced access to certain foods that are sources of these nutrients, such as fish [34]. The unstable correlations observed for polyunsaturated fat and omega 3 fatty acids may also be explained by the fact that the HSFFQ was designed specifically for the WIC population to assess intakes of seven nutrients (protein, calcium, iron, zinc, and vitamins A, B6, and C) but not fat, whereas the Minnesota Nutrient Data System used for the diet recalls was designed to assess fat content. Another limitation of the HSFFQ is its inability to estimate absolute nutrient intake. Because the HSFFQ has a limited number of items and minimal information about portion size, it is not intended to provide accurate estimates of absolute intake; therefore, our findings apply only to its use as a research tool for classifying these women according to their relative nutritional intake [13]. It is also possible that the correlations between nutrient intakes from the HSFFQ and the diet recalls could be overestimated, because the two methods have correlated sources of error (i.e., both rely on memory) and the process of completing the recalls prior to the HSFFQ could have improved awareness of food intake [13].

## Conclusion

The present study complements past and recent research on the reproducibility and validity of the Harvard Service FFQ. Based on our results, the HSFFQ may be a useful research tool for assessing relative nutritional intake among low-income American Indian and Caucasian women during different stages of pregnancy. It could provide important information for epidemiologic studies of pregnancy diet in relation to fetal outcomes and also serve as a starting point for identifying groups of women who may benefit most from nutritional interventions. Further research is needed to evaluate the validity of FFQs for the assessment of nutrients with high day-to-day variation and for particular subgroups of pregnant women.

## Competing interests

The author(s) declare that they have no competing interests.

## Authors' contributions

HJB conducted the data analysis and drafted the manuscript with REB. REB coordinated the data collection, performed preliminary analyses, and with HJB drafted the manuscript. HRHR ran all nutrient analyses and coordinated the verification of the nutritional data. JL recruited North Dakota WIC clinics as study sites and provided insight into the study population. JDG and CWS contributed to the concept and design of the study. GAC contributed to the concept and design of the study and obtained the funding. All authors read and approved the final manuscript.

## Pre-publication history

The pre-publication history for this paper can be accessed here:


